# Two-step deep-learning identification of heel keypoints from video-recorded gait

**DOI:** 10.1007/s11517-024-03189-7

**Published:** 2024-09-18

**Authors:** Kjartan Halvorsen, Wei Peng, Fredrik Olsson, Anna Cristina Åberg

**Affiliations:** 1 School of Health and Welfare, Dalarna University, Falun, Sweden; 2 Department of Public Health and Caring Sciences, Uppsala University, Uppsala, Sweden; 3 Stardots AB, Uppsala, Sweden

**Keywords:** Gait analysis, Marker-less motion capture, Convolutional neural networks

## Abstract

**Abstract:**

Accurate and fast extraction of step parameters from video recordings of gait allows for richer information to be obtained from clinical tests such as Timed Up and Go. Current deep-learning methods are promising, but lack in accuracy for many clinical use cases. Extracting step parameters will often depend on extracted landmarks (keypoints) on the feet. We hypothesize that such keypoints can be determined with an accuracy relevant for clinical practice from video recordings by combining an existing general-purpose pose estimation method (OpenPose) with custom convolutional neural networks (convnets) specifically trained to identify keypoints on the heel. The combined method finds keypoints on the posterior and lateral aspects of the heel of the foot in side-view and frontal-view images from which step length and step width can be determined for calibrated cameras. Six different candidate convnets were evaluated, combining three different standard architectures as networks for feature extraction (backbone), and with two different networks for predicting keypoints on the heel (head networks). Using transfer learning, the backbone networks were pre-trained on the ImageNet dataset, and the combined networks (backbone + head) were fine-tuned on data from 184 trials of older, unimpaired adults. The data was recorded at three different locations and consisted of 193 k side-view images and 110 k frontal-view images. We evaluated the six different models using the absolute distance on the floor between predicted keypoints and manually labelled keypoints. For the best-performing convnet, the median error was 0.55 cm and the 75% quartile was below 1.26 cm using data from the side-view camera. The predictions are overall accurate, but show some outliers. The results indicate potential for future clinical use by automating a key step in marker-less gait parameter extraction.

**Graphical abstract:**

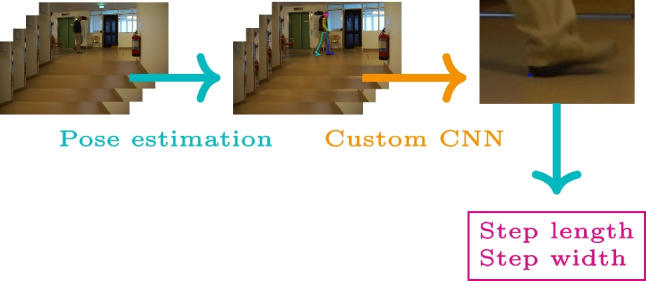

## Introduction

Motion analysis using recordings with regular video cameras or cell phones and without the use of markers attached to the subject is attractive, since it opens up for much wider use of motion capture and gait analysis, compared to laboratory-grade motion capture systems. Within the Uppsala-Dalarna Dementia and Gait project (UDDGait™), Timed Up and Go (TUG) is combined with a cognitive/verbal task (TUG dual-task, TUGdt) [6] and has been developed as a way of identifying cognitive impairment and dementia. The tests are performed in clinical settings, which favors data acquisition methods that are easy to set up and unobtrusive for the participant. The focus in this work is therefore on extracting step parameters from video recordings.

Marker-less motion capture can be achieved with the use of deep-learning methods. Image location of body keypoints in regular images can be obtained by the use of convolutional neural networks (convnets) and can form the basis for human movement analysis. Several marker-less methods have been proposed for extracting gait parameters from video recordings, making use of the open-source software OpenPose [5] and similar, for instance, [9, 11, 22]. OpenPose has also been used for human movement analysis in the context of sprinting [17] and fall detection [7]. In these works, the anatomical landmarks corresponding to the OpenPose keypoints were used for direct calculation of gait or movement parameters, except for the paper [12], in which the OpenPose keypoints were used as feature vectors (inputs) to train a custom convnet regression model. The use of OpenPose and similar models (e.g., AlphaPose [14, 23]) shows great potential for human movement analysis. However, the accuracy of the identified keypoints is not yet sufficient for detailed movement analysis relevant for many clinical purposes. In [22], the authors compare gait parameters obtained from keypoints identified by OpenPose with camera-based 3D motion capture, reporting average absolute errors of 0.02 s for gait-phase durations and 5 cm for step length. The errors were reduced to 0.01 s and 1.8 cm, respectively, when individual averages were calculated, indicating the presence of random measurement errors in the OpenPose keypoints. An investigation of the accuracy of 3D keypoint locations reconstructed from 2D image keypoints obtained by OpenPose showed mean absolute errors between 2–5 cm [16]. It has been suggested that gait analysis based directly on keypoint locations from OpenPose can be improved by including more cameras [8], but this adds to the complexity of the data acquisition. Although the accuracy of the keypoint locations from OpenPose is too low for calculating clinically meaningful joint angles and gait parameters, the keypoint trajectories can provide intermediate data on the human movement such that sequences and regions of interest in the image can easily be found automatically.

We have previously proposed a semi-automatic method for extracting gait parameters [2, 3], in which approximate body keypoints are obtained from OpenPose and are used to find time intervals of interest (gait), approximate events (heel-strikes and toe-offs), and regions of interest (feet) in the video. Manual correction of events and keypoints on the heel is currently still needed, which makes the extraction of gait parameters time-consuming. The validity (mean differences from 6.4 to 13.0% compared to marker-based motion capture) and reliability (ICC> 0.90) of the manual steps of this method is good [2, 3], which is of direct importance for the deep-learning method presented in this article, since methods are trained and evaluated against manually labelled data.

The focus of the current work was to investigate the ability of convnets to determine keypoints on the heel from marker-less video images with sufficient accuracy to be of clinical relevance. In particular, we aimed to determine contact points of the heel with the ground. These points are, by definition, on the floor plane, and so by calibrating the floor plane, the 3D locations can be determined from a single video camera. In a subsequent step, the 3D heel point locations can be used to obtain step length and step width. This article focuses on the accuracy in detecting the heel contact points. We evaluated three pre-trained convnet architectures that have been shown to perform well for keypoint detection and pose estimation: VGG19 [21], ResNext50 [28], and MobileNetV2 [19]. Each of the three networks was used for feature extraction (backbone network) and combined with two different networks (head networks) for predicting the heel keypoint. This study is a step towards a fully automatic and accurate marker-less gait analysis.

## Materials and methods

### Data

The data sample consisted of trials from 184 participants (age 40–90, 45% women) recruited for tests at three different locations in Sweden and Finland: The Swedish School of Sport and Health Sciences, Stockholm; Uppsala University Hospital, Uppsala; and Åland University of Applied Sciences, Mariehamn. The study was approved by the Regional Ethical Review Board in Uppsala (dnr. 2010/097/1;2) and the Swedish Ethical Review Authority (dnr. 2019-03282).

### Data collection

**Fig. 1:**
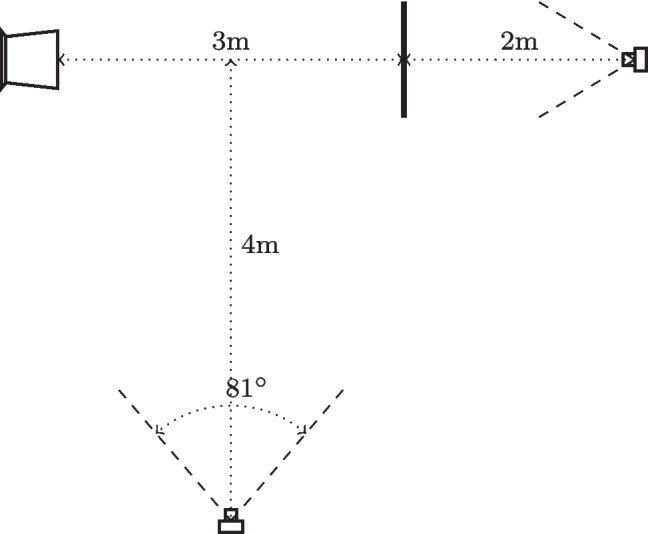
Experimental setup. Two cameras were used, recording the TUG test from the side and from the front

The participants performed TUG tests, consisting of initially sitting on a chair, rising up, walking 3 m straight ahead passing a line marked on the floor, turning around, returning to the chair, and sitting down again. Each participant performed one regular TUG and two or three different types of TUG trials with concurrent verbal task, according to the established protocol [1, 2, 6] for the UDDGait™study. The participant’s performance was documented using two video cameras (Sony NEX-5T) on tripods; one placed 2 m in front of the line where the participant turned and the other 4 m to the side of the line. The cameras’ field of view was 81$^{\circ }$, and high-definition videos (1080p) were recorded at a frame rate of 25 Hz. See Fig. 1. The cameras were synchronized by either an audible signal (double-clap, used in one location) or by a digital clock visible in both camera views (used in the other two locations).

**Fig. 2:**
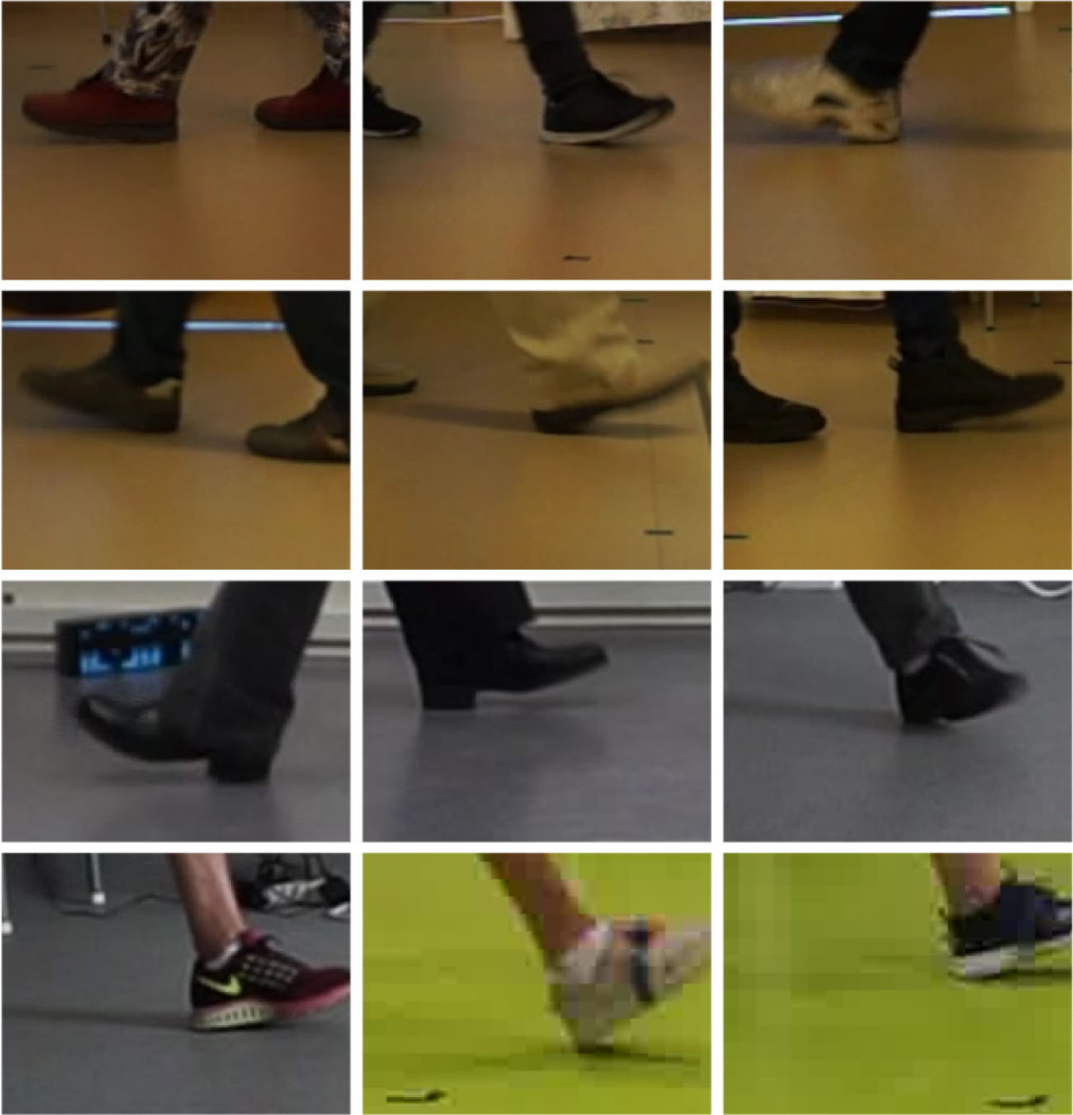
Examples of cropped foot images

Five to eight points (differing between the distinct test locations) on the floor were marked with black tape at known locations in a frame of reference with one coordinate axis (*x*) pointing from the chair to the 3-m line, and another, perpendicular coordinate axis (*y*) pointing to the left as seen when sitting on the chair. For each video camera, the image coordinates of the floor markings were determined, from which a homography was computed using the OpenCV library [4]. This provided a mapping between the image plane and the floor plane, by which keypoints at the contact between foot and floor detected in the image could be projected onto the floor to obtain the corresponding 3D world coordinates.

### Data processing

The video recording was split into a sequence of images using ffmpeg [25] and subsequently processed with OpenPose [5] to obtain 25 body keypoints for each video frame. The trajectories of the detected keypoints on the feet served to identify parts of the video containing gait and to give an initial estimate of the heel-strike event in the gait sequences. The heel-strike event was approximated with the frame showing a peak in the absolute heel-to-heel distance, after which the approximate heel-strike event was corrected by visual inspection [2, 3]. In the heel-strike frame, the foot centroid, i.e., the center of the “ankle”-, the “big-toe”-, and the “heel” keypoints obtained from OpenPose was calculated and used to obtain images of the feet only. See Fig. 2.

**Fig. 3:**
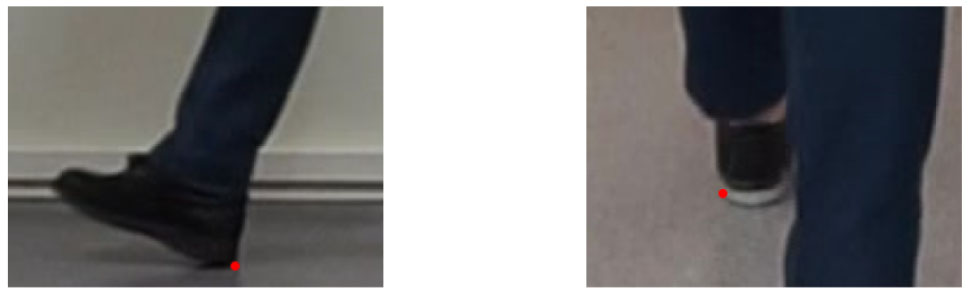
Examples of labelled images. The red point is the heel keypoint labeled by human

To obtain labelled image data, cropped images containing the foot at heel strike were scaled up by a factor of four. In this cropped and scaled image, the heel keypoint was identified by visual inspection as the most posterior point on the heel that has contact with the floor (side view) or the most lateral point of the heel that has contact with the floor (frontal view). See Fig. 3. The procedure was repeated for the next two frames (side view) or the next frame (frontal view) in the sequence, in order to obtain more image data.

The complete dataset contained a total of 4824 side and 2756 frontal foot images. The dataset was augmented by 20 random translations horizontally and vertically according to a uniform distribution with a width of 60 pixels, and flipping of each translated image. With augmentation, the size of the side foot dataset was 192 k, and the size of the frontal dataset was 110 k. All images in the dataset had a width of 160 pixels and a height of 120 pixels.

The 184 subjects/trials were randomly assigned for training (157), validation (9), and test (18). The same assignation was used for all training and evaluation of the different models. The validation data was used for tuning the hyper-parameters of the models, whereas the test data was used only in the final step for generating the performance results presented in this article.

### Architecture, training, and evaluation of the convolutional neural networks

Convolutional neural networks (convnets) take images as input and generate a prediction at the output. The three alternative architectures for the backbone network considered in this work were all designed for the ImageNet [10] dataset, and thus their respective output layer has 1000 nodes, corresponding to the 1000 classes of objects defined in ImageNet. Specifically, their output is a vector of 1000 values corresponding to the predicted probability that the image at the input contains an object belonging to one of the classes. The first, successive layers of deep neural networks extract features from basic- to higher-level from images [13]. These features are represented as three-dimensional arrays (tensors) where the dimensions are width, height, and number of channels. In the last few layers (the number depending on the particular network architecture), the output is transformed into one-dimensional arrays (vectors), which encodes the classification output of the network. It is common to refer to these last layers as the *classification layers* and the part of the network that comes before as the *feature-extraction layers*.

The architectures we propose in this work modify the classification layers, by combining part of the pre-trained architectures (backbone networks) with new classification layers designed for the task at hand to predict a single keypoint on the heel. This is a regression task and not a classification task. We compared two different classification layers, or *head networks*, that are attached to the output of the backbone networks, and refer to these as *regression head network* and *heatmap head network*. We considered three different convnets for the backbone: VGG19 [21], ResNext50 [28], and MobileNetV2 [19], chosen because of well-known performance in pose estimation [20, 24, 26].

In the case of the regression head network, this consisted of a fully connected linear layer with input size 1000 and output size 2, providing the normalized (to the image size) *x* and *y* image coordinates of the predicted heel keypoint. This layer was attached directly to the output of the backbone network. In the case of the heatmap head, we kept only the feature-extraction part of the backbone networks (VGG19, first 16 of 19 layers; MobileNetv2, all layers except final two; and ResNext50, all layers except final two). This backbone network (with pre-trained weights) added five layers of transposed convolutional layers that increase the spatial resolution and one final convolutional layer to bring the number of channels down to one (since only a single keypoint is predicted). The number of transposed convolutional layers was chosen so that the final array (tensor) had width and height similar to the input image. Due to the specific architecture of the backbone convnets, it is not always possible to get back the exact same width and height as the input image. The predicted keypoint image location was calculated as the mean position using a 13 $\times$ 13 neighborhood around the pixel with the maximum value in the heatmap.

Networks were trained separately for the side-view and the frontal-view images, since the appearance of the foot is very different in the two views. The cropped image of the foot formed the input to the network, and an estimate of the heel keypoint in the image formed the output. The convnets were trained using the mean-square error of the image point prediction as loss function. The optimization algorithm used for training was stochastic gradient descent (SGD), with a momentum of 0.9. All the models were trained for 20 epochs. The batch size and learning rate were tuned individually for each model, by evaluating the performance using the validation data. The batch size was varied between 8 and 12, and the learning rate was between $10^{-3}$ and $10^{-2}$. The learning rate was decayed by 0.1 after 10 epochs. In order to avoid over-fitting, a weight decay of 0.0001 was used in the SGD optimizer to penalize large weights. Early stopping with a patience of 4 was also introduced, such that the training process would stop if the validation loss did not decrease for 4 epochs. During training, dropout was implemented with a dropout rate of 0.5.

**Fig. 4:**
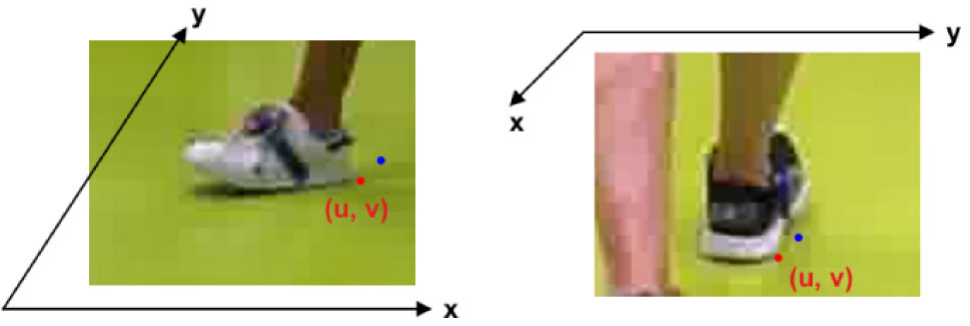
The blue and red points are the predicted and labeled heel keypoints, respectively. The axis lines show the directions of the reference frame on the floor. Examples with a large (not typical) discrepancy between labeled and predicted keypoint are shown

**Fig. 5:**
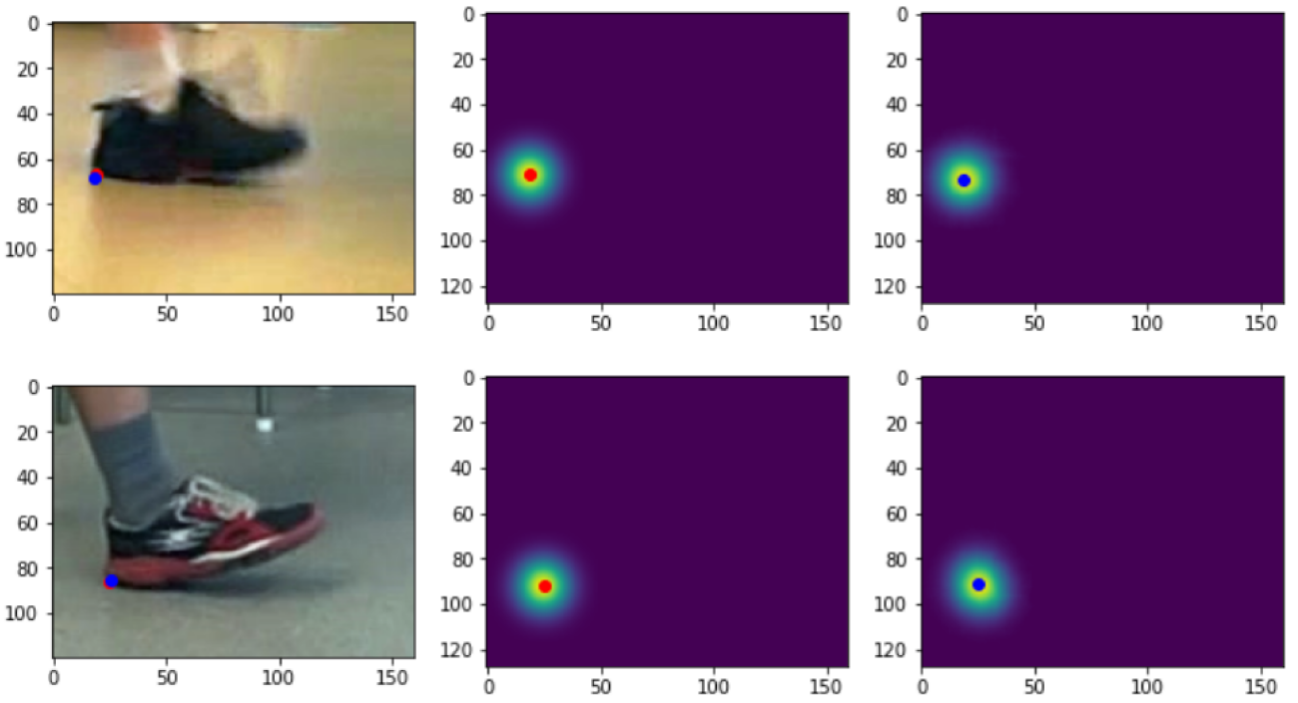
Example of heatmaps generated from the labelled keypoint (middle) and predicted heatmap (right). The red dot is the labelled keypoint, and the blue is the predicted one

We made use of transfer learning, which means fine-tuning the parameters of a model that has previously been trained on a larger dataset, in this case, the ImageNet [10] dataset. It is well-established that transfer learning gives better performance than training from scratch [29], in particular, if the new dataset is small and the model is to be applied to images of individuals that are not present in the training data [15].

For the final evaluation using the test data, the model performance was evaluated by calculating the prediction error along one single axis on the floor plane, in world coordinates, since this is the relevant error when using the method to extract the step parameters *step length* and *step width*.

Figure 4 shows the world-coordinate system overlayed on cropped foot images. For a side-view image, the evaluation considered the absolute error between the manually labelled heel point and the point predicted by the convnet in the direction of the floor *x*-axis (the direction of walking). Similarly, for a frontal-view image, the evaluation considered the absolute errors in the direction of the floor *y*-axis. An example with a relatively large error is shown for clarity.

**Fig. 6:**
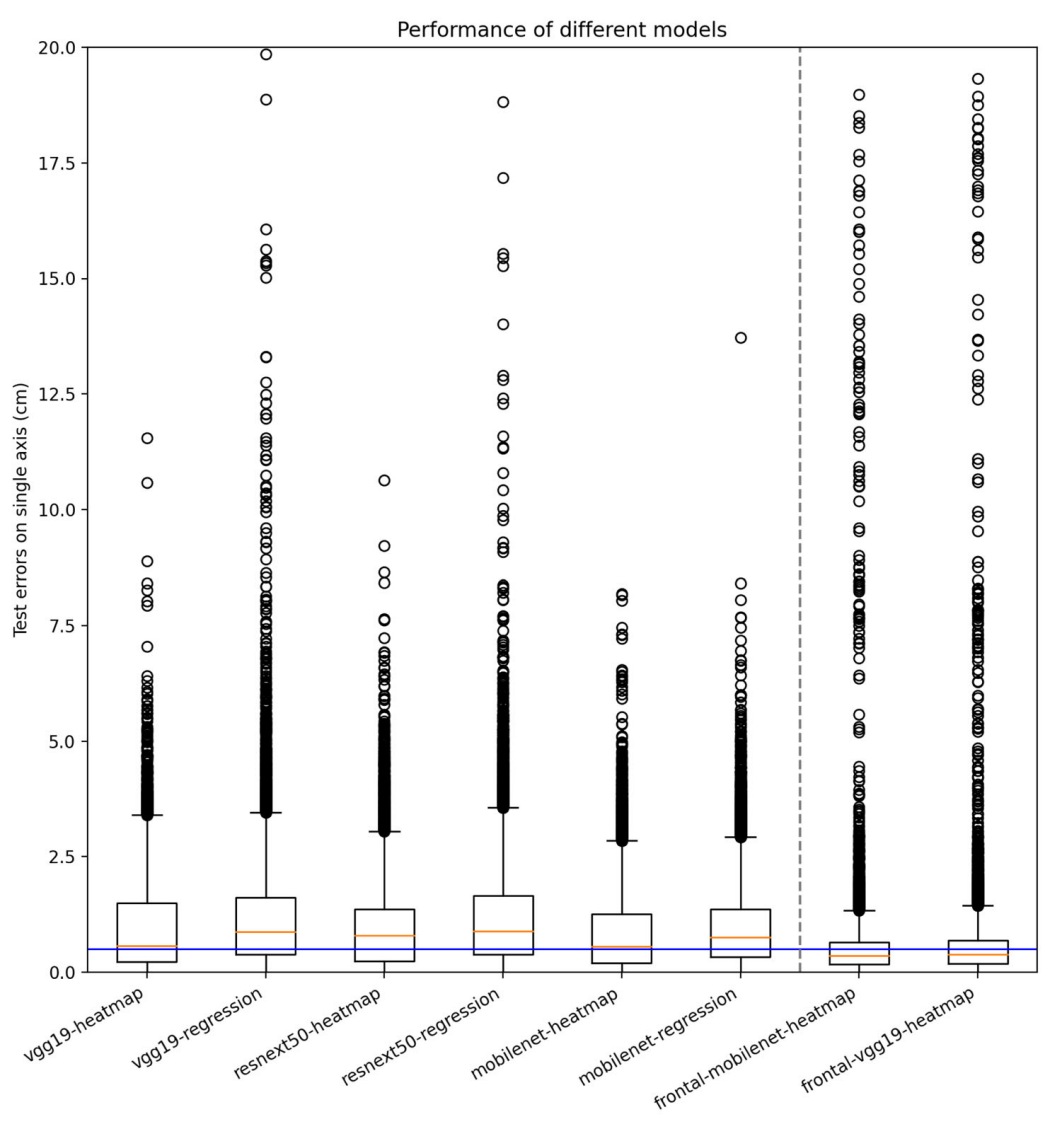
Errors in relevant direction on the floor for the test data. The left six models were trained on the side-view images. The two models to the right were trained on frontal-view images. In the box plot, the box extends from the first quartile ($Q_1$) to the third quartile ($Q_3$), with a line at the median (the orange line). The height of the box, i.e., $Q_3-Q_1$, is the inter-quartile range (IQR). The whiskers end at extremum values, with maximum at $Q_3+1.5\times \text {IQR}$ and minimum at $Q_1-1.5\times \text {IQR}$. The individual data points outside the whiskers are the outliers. The individual data points outside the whiskers are the outliers. The ends of the whiskers are the maximum and minimum respectively excluding outliers. For reference, the error of 0.5 cm is marked with a blue line in the figure

**Table 1:** Summary statistics for the test errors

Model	#Pars	Min	Max	Median	Q1	Q3	IQR	Outl.
vgg19-heatmap	23.6	0.000	42.717	0.574	0.231	1.499	1.268	0.056
vgg19-regression	143.7	0.000	29.869	0.868	0.381	1.612	1.230	0.082
resnext50-heatmap	30.1	0.000	38.535	0.788	0.239	1.364	1.125	0.073
resnext50-regression	29.0	0.000	18.819	0.882	0.391	1.658	1.268	0.081
mobilenet-heatmap	4.1	0.000	8.186	0.550	0.196	1.258	1.061	0.082
mobilenet-regression	3.5	0.000	13.715	0.751	0.332	1.369	1.037	0.075
front-vgg19-heatmap	23.6	0.000	31.169	0.379	0.182	0.686	0.504	0.104
front-mobilenet-heatmap	4.1	0.000	29.081	0.358	0.176	0.643	0.467	0.095

The number of parameters (#Pars) is given in millions. Minimum value, maximum value, percentiles, and intra-quartile range are given in centimeters. The outlier value is the ratio of the number of outliers to the number of observations

For the side-view images, all six models were evaluated. Based on the result from the side-view models, the best two models were chosen for training with the frontal-view images.

All the models were implemented in PyTorch [18], and all the experiments were run on a workstation with CPU Intel Xeon W-2223 and GPU Nvidia RTX 2080Ti.

## Results

Figure 5 shows an example of heatmaps generated from the labelled image and heatmaps predicted by the convnet. The red dot is the labelled keypoint, and the blue is the estimated position based on the predicted heatmap.

Figure 6 shows the distribution of errors, and Table 1 provides numbers on the performance of all the eight models, of which six models were trained on the side-view images and two were trained on the frontal-view images. The errors for all test images are presented in the form of box plots with median, inter-quartile range, maximum value, and outliers marked.

## Discussion

The results of Fig. 6 and Table 1 show that all the tested convnets are capable of locating the heel keypoint in both the side-view and frontal-view images with a median error of less than 0.9 cm and third-quartile error of less than 1.7 cm. For the side-view, the MobileNetV2 model is the most promising, with a median error of 0.55 cm, third-quartile error of 1.3 cm, and maximum error of 8.2 cm. Notably, this model also has the fewest parameters (4.1 mill), thus providing a lightweight (relatively low memory requirements) and well-performing model. Comparing the use of a heatmap head vs regression head, we note that the convnets using heatmaps had a smaller median error, although the variance (measured as IQR) and the fraction of outliers are similar. It can also be observed that the median error is smaller in the frontal view; however, we contribute this mostly to the fact that the feet are closer to the camera for several steps, and thus, compared to the side-view image, a similar error in image distance translates to a smaller error when projected onto the floor.

Current literature on the accuracy of deep-learning methods for motion capture indicates that the accuracy reported here is an improvement over directly using keypoints provided by deep-learning pose estimation methods. In a recent article [27], several pose estimation methods for measuring gait kinematics were compared, among which OpenPose performed best. Errors in joint angles were reported, and it was concluded that pose estimation from video can be a useful alternative to conventional motion capture, when such systems are not available and when high accuracy is not required. The accuracy of OpenPose has been investigated for 3D reconstruction using five cameras [16], where it was found that in walking the mean average error in the ankle keypoint was about 3 cm in the anterior-posterior direction and 1 cm in the medio-lateral direction. Although the results are not directly comparable with the accuracy of our method, they indicate that the accuracy obtained with one camera and the convnets proposed in this article is better than the use of OpenPose keypoints directly and with five cameras. It should be noted that the accuracy of manually labelling the heel keypoints to obtain step parameters reported in [2] is a substantial improvement over the direct use of keypoints detected by OpenPose, as investigated in the article [22], which reported a value for the 95% limit of agreement between reference system (optical motion capture) and OpenPose of 12 cm for step length, compared to 2 cm reported when using manually labelled keypoints with the method in [2]. We did not attempt to compare results on step length and step width obtained with the proposed method against step parameters obtained by openpose on the same data, for the reason that the necessary steps in the methods would differ substantially, and it would remain unclear what causes differences in the results.

The level of accuracy indicated by the results means that the keypoint locations provided by the proposed deep-learning method can be used to calculate step length and step width from video recordings with meaningful accuracy. One should keep in mind, though, that the models are trained with—and the errors are measured against—keypoints labelled by humans, which in themselves have limited accuracy. The particular labelled image data used in this work were obtained using a methodology that has been tested for validity and inter- and intra-rater reliability, with good results [2]. Important aspects in obtaining consistent and valid results when labelling images are having clear definitions of what is to be marked and using images with good resolution that can be zoomed.

We chose to train separate models for the side-view and frontal-view images, with the motivation that the foot looks different as seen from the side or from the front/back, and the definition of the heel keypoint that the convnets try to predict is also different in the two views: posterior-inferior point in the side view and lateral-inferior point in the frontal view.

The performance of the convnets presented here is obtained with a comparably small amount of training data. The reason that this is possible is by the use of transfer learning, where the model parameters are initialized to their values from training on the ImageNet data set. Furthermore, the problem is constrained to finding a single point on cropped images of feet only, thus reducing greatly the variation in the image data, compared to the general images of humans that OpenPose and similar pose estimation methods are able to deal with. As with all deep-learning methods, the performance of the methods on a new image depends on the similarities between the new image and the set of images that the method has been trained on. The image data used in this work consisted of data from 184 individuals at three different locations. Hence, there is some variation in the footwear, in the texture and color of the floor, and in the lighting conditions. The images contained the whole foot, with only parts of the other foot visible. It is to be expected that the performance of the method will decrease when confronted with a new set of images that differ significantly from the training set. The strategy we propose to deal with this is to add a step of visual inspection of the result provided by the convnet, in particular in new cases which are assumed to differ from the training data set, such as from a new location, or from abnormal gait. Following the visual inspection, the user may correct the heel point, and this corrected location together with the image is stored to build up new training data for re-training of the model. Over time, we expect the performance to become more and more robust, to the point when the visual inspection can be disposed of.

The trained convnets are of similar complexity as the convnets used as backbones; some final layers are removed, and some are added. In terms of number of parameters, the models vary (see Table 1) and some have more, and some are less than OpenPose (26 mill). Running a trained model to obtain a predicted heel point is in general fast, around 10 ms on a workstation with a CPU Intel Xeon W-2223 and GPU RTX 2080Ti.

The aim is to develop a fully automated convnet-based method for analyzing the TUG video data. Further development is needed to reach this goal, and presently, several manual steps are required in the complete procedure. The most important next step in the development is to develop automatic detection of the gait events, from which the images of the feet can be extracted and the method proposed here applied.

## Conclusion

There are two important contributions that this article offers. Firstly, we show that the accuracy of identifying heel keypoints automatically and without markers can be improved over the much-used OpenPose method, even with a relatively small amount of data, by utilizing transfer learning. Secondly, we present a novel, practical two-step method for analyzing video recordings of TUG experiments, using rough estimates of keypoints provided by OpenPose in a first step, and then a custom-trained convnet to determine the important heel point. The results are promising from a clinical point of view since they provide a basis for the development of an efficient program for clinical practice, which may prove a useful assessment tool for the early identification of dementia disorder in various healthcare settings. This, in turn, has the potential to improve the pathway for people with cognitive impairment and their carers, as it would allow for the initiation of earlier interventions to prevent and/or delay symptoms and maintain quality of life, as well as possibly increase the efficiency of care delivery.

## Data Availability

The material analyzed during the current study is not publicly available due to its content of sensitive personal data. Datasets generated may be available on request, after ethical considerations.
